# Pervasiveness of Parasites in Pollinators

**DOI:** 10.1371/journal.pone.0030641

**Published:** 2012-01-26

**Authors:** Sophie E. F. Evison, Katherine E. Roberts, Lynn Laurenson, Stéphane Pietravalle, Jeffrey Hui, Jacobus C. Biesmeijer, Judith E. Smith, Giles Budge, William O. H. Hughes

**Affiliations:** 1 Institute of Integrative and Comparative Biology, University of Leeds, Leeds, United Kingdom; 2 National Bee Unit, Food and Environment Research Agency, York, United Kingdom; 3 Center for Infection and Immunity, Columbia University, New York, New York, United States of America; 4 Netherlands Centre for Biodiversity NCB Naturalis, Leiden, The Netherlands; 5 School of Environment and Life Sciences, University of Salford, Salford, United Kingdom; Ghent University, Belgium

## Abstract

Many pollinator populations are declining, with large economic and ecological implications. Parasites are known to be an important factor in the some of the population declines of honey bees and bumblebees, but little is known about the parasites afflicting most other pollinators, or the extent of interspecific transmission or vectoring of parasites. Here we carry out a preliminary screening of pollinators (honey bees, five species of bumblebee, three species of wasp, four species of hoverfly and three genera of other bees) in the UK for parasites. We used molecular methods to screen for six honey bee viruses, *Ascosphaera* fungi, Microsporidia, and *Wolbachia* intracellular bacteria. We aimed simply to detect the presence of the parasites, encompassing vectoring as well as actual infections. Many pollinators of all types were positive for *Ascosphaera* fungi, while Microsporidia were rarer, being most frequently found in bumblebees. We also detected that most pollinators were positive for *Wolbachia*, most probably indicating infection with this intracellular symbiont, and raising the possibility that it may be an important factor in influencing host sex ratios or fitness in a diversity of pollinators. Importantly, we found that about a third of bumblebees (*Bombus pascuorum* and *Bombus terrestris*) and a third of wasps (*Vespula vulgaris*), as well as all honey bees, were positive for deformed wing virus, but that this virus was not present in other pollinators. Deformed wing virus therefore does not appear to be a general parasite of pollinators, but does interact significantly with at least three species of bumblebee and wasp. Further work is needed to establish the identity of some of the parasites, their spatiotemporal variation, and whether they are infecting the various pollinator species or being vectored. However, these results provide a first insight into the diversity, and potential exchange, of parasites in pollinator communities.

## Introduction

Pollinators are of great ecological and economic importance. They pollinate a wide variety of crops with an estimated global value of $153 billion pa [1], [2], and are also essential for the reproduction of at least two thirds of flowering plant species, including many which are now endangered [3], [4], [5], [6]. Although much attention has focused on managed populations of honey bees, pollinators include a diversity of insects, with the main groups in temperate areas being bumblebees, social wasps, hoverflies and solitary bees. However, many pollinator populations appear to be decreasing, although declines are by no means universal [7], [8]. The species richness of bees in the UK and Holland has decreased substantially [9], [10], and around a third of honey bee colonies in the US have been lost each year since 2006, in part due to a syndrome termed ‘Colony Collapse Disorder’ [11]. Of the UK's 25 bumblebee species, 3 are now extinct, 7 are threatened and 15 have undergone major range contractions in recent years [10], while North American bumblebees are also declining in abundance and species richness [12], [13]. There are a multitude of factors that are responsible for this, including land use change, pesticide exposure, reductions in population genetic diversity and climate change [14], [15], [16].

One factor that may be particularly important in pollinator declines is parasites. Parasites are a key selection pressure for most organisms, including insects. Some insect parasites are highly virulent and cause obvious disease symptoms, such as the obligate killer fungal parasites *Metarhizium* and *Ascosphaera* [17], [18], [19], [20]. Other parasites are far less obvious, such as *Wolbachia* intracellular bacteria, which are widespread in insects and can have a major impact on host fitness by manipulating host sex ratios or negatively affecting host survival [21], [22]. The parasites of honey bees are relatively well known and have been implicated in the recent colony losses seen in the US and elsewhere [14], [23], [24], [25], [26]. They include apparently long-established host-parasite relationships such as the *Ascosphaera apis* fungal parasite which causes chalkbrood disease, the microsporidian *Nosema apis* which causes dysentery, and many viruses [27]. However, they are also characterised by a number of emerging parasites, the appearance of which can have large impacts on honey bee populations, such as the microsporidian *Nosema ceranae* in Spain and Portugal [25], [28], [29], [30]. Bumblebees too may be infected by *N. ceranae* [31], as well as suffering from their own microsporidian, *Nosema bombi*, which can have major effects on their fitness [32], [33], [34].

However, our knowledge of the parasites that afflict other pollinators is far more limited. The economically important alfalfa leaf-cutter bee (*Megachile rotundata*) is well known to suffer from *Ascosphaera* fungi [35], [36], and the solitary bee *Andrena scotica* has been found to have high prevalence infections by the *Antonospora scoticae* microsporidian [37], [38], but nothing is known of the parasites of the vast majority of pollinators. In addition, the shared use of flowers by communities of pollinator species represents a significant opportunity from the perspective of a parasite for interspecific transmission or at least vectoring (i.e. transport of a parasite without infection). The discoveries of *Nosema ceranae* in Argentinean bumblebees [31], deformed wing virus (DWV) in German *Bombus pascuorum* and commercial *Bombus terrestris* [39], and of acute bee paralysis virus (ABPV) in UK bumblebees [40], suggest that even intergeneric pathogen spillover can occur. Most recently, molecular screening of bees and wasps collected near apiaries in the USA found that many were positive for various honey bee viruses [41], although the limited sampling effort did not provide any information on prevalence. Far more information is therefore needed on the frequency of parasites in pollinators, including those known to cause disease in honey bees.

Here we carry out a preliminary examination of the occurrence of parasites in a variety of pollinators from across the UK. We collected samples of social wasps, bumblebees, hoverflies, honey bees and other bees (all of which pollinate flowers [9], [42], [43], [44]) and used molecular methods to screen them for the presence of the six most common honey bee viruses, *Ascosphaera* fungi, Microsporidia, and the *Wolbachia* intracellular bacterium.

## Results

**Figure 1:**
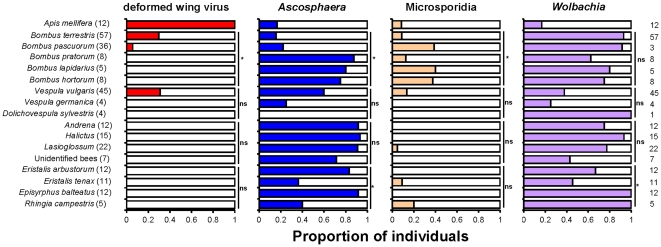
Incidence of parasites in pollinators. Overall proportions of honey bees (*Apis mellifera*), five species of bumblebees (*Bombus* spp.), three species of wasps (*Vespula vulgaris*, *V. germanica*, *Dolichovespula sylvestris*), three genera of other bees (*Andrena*, *Halcitus*, *Lasioglossum*; there were also some that could not be identified) and four species of hoverflies (*Eristalis arbustorum*, *E. tenax*, *Episyrphus balteatus*, *Rhingia campestris*) which were positive (coloured) or negative (white) for the deformed wing virus (DWV), *Ascosphaera* fungus, Microsporidia and *Wolbachia* parasites. Sample sizes for each host are given in parentheses. Significant differences in incidence between species within each of the five host types (honey bees, bumblebees, wasps, other bees, hoverflies) are indicated to the right of the relevant bars (* = *P*<0.05; ns = *P*>0.05).

Out of 325 pollinator samples collected, DNA and RNA was successfully extracted from 272 individuals and amplified in both conventional and real-time PCRs, based on the internal control gene (Table S1). Pollinators were found to be frequently positive for *Wolbachia* and *Ascosphaera*, and less frequently for Microsporidia and DWV parasites (Figure 1). One *Vespula vulgaris* wasp was positive for black queen cell virus and another for sacbrood virus. ABPV, chronic bee paralysis virus and Israeli acute paralysis virus were not detected in any samples. The *CoxA* sequences obtained from five hoverflies and 7 bees were all confirmed to be *Wolbachia*. Eight bumblebees, three wasps and one *Lasioglossum* bee were sequenced for the *V1f/530r* microsporidian gene, with the bumblebee sequences all matching most closely (96–100%) that of *Nosema bombi*. The microsporidians from wasps most closely matched *Nosema bombi* (95–97%), while that from the *Lasioglossum* bee had no strong match. The *Ascosphaera* sequences of four bees were examined and two (one *Andrena* and one *Halictus*) closely matched that of *Ascosphaera apis* (98%), while the other two (one *Lasioglossum* and one *Halictus*) had no strong match.

The pollinator types differed significantly in the numbers of individuals that were positive for DWV (χ^2^ = 82, df = 4, *P*<0.001), *Ascosphaera* (χ^2^ = 69.8, df = 4, P<0.001), Microsporidia (χ^2^ = 19.4, df = 4, *P* = 0.001) and *Wolbachia* (χ^2^ = 60.3, df = 4, *P*<0.001). All honey bees were positive for DWV, as were about a third of *Vespula vulgaris* wasps, while bumblebee species interestingly varied significantly, with a third of *Bombus terrestris* bumblebees and also a small number of *B. pascuorum* being positive, but all other species being entirely negative (Figure 1). *Ascosphaera* was common in *Andrena*, *Halictus* and *Lasioglossum* bees, of moderate frequency in wasps and honey bees, while its incidence varied between both bumblebee species and hoverfly species (Figure 1). Microsporidia were in general relatively rare, being found at the greatest frequency in bumblebees where a fifth of samples overall were positive and incidence differed between species (Figure 1). *Wolbachia* was very common in bumblebees, *Andrena*, *Halictus*, and *Lasioglossum* bees, but infected only 17% of honey bees, and differed in incidence between the four hoverfly species (Figure 1).

## Discussion

Our use of molecular methods revealed that many pollinators were positive for the parasites that we screened for. Both *Ascosphaera* and *Wolbachia* were very prevalent, being found in most pollinators of all types. Microsporidia were much rarer, mainly being found in bumblebees. We found no evidence of acute bee paralysis virus, chronic bee paralysis virus or Israeli acute paralysis virus in any of the pollinators, and only found a single *V. vulgaris* wasp positive for sacbrood virus and another for black queen cell virus. However, we did find that a third of *Bombus terrestris* and a third of *Vespula vulgaris* wasps were positive for deformed wing virus, as well as all of the honey bees in our sample. Inefficiencies in the Chelex extraction method used [45], [46], mean that true levels of the viruses may be greater still.

All of the pollinator taxa we examined included individuals that were positive for *Wolbachia*. As *Wolbachia* are intracellular bacteria, the positive amplification of it means these individuals were most probably infected by the bacteria rather than vectoring them. *Wolbachia* is a widespread parasite of insects [22], including many ants [47], [48], [49], but has been surprisingly little investigated in social bees and wasps. It has previously been found in the *Apis mellifera scutellata* and *A. m. capensis* African subspecies of the Western honey bee [50], [51], [52], and in *Osmia* (Megachilidae), *Rediviva* (Melittidae), *Agapostemon* (Halictidae), *Colletes* (Colletidae) and *Diadasia* (Apidae) solitary bees [53], [54]. Our results therefore confirm that *Wolbachia* infects honey bees, and also expand its host range to include bumblebees, *Andrena* (Andrenidae), *Halictus* (Halictidae) and *Lasioglossum* (Halictidae) bees, as well as wasps and hoverflies. In other insects, *Wolbachia* can have profound effects on host sex ratios, as well as negatively or positively affecting other aspects of host fitness [22], [55], [56], [57], [58], [59]. It will therefore be important to discover what effects *Wolbachia* has on its pollinator hosts.

The *Ascosphaera* fungi, which cause chalkbrood disease, were found very commonly in many of the pollinator taxa that we sampled. *Ascosphaera* has previously only been recorded as a parasite of honey bees, *Megachile*, *Osmia* and *Coelioxys* megachilid solitary bees [18], [60], [61], [62], [63], and as an apparently non-pathogenic symbiont of *Nomia* (Halictidae) solitary bees [64]. In all cases it specifically infects the larval life-stage of the host insect and has not previously been recorded from the adult life-stage that made up our samples. It is unlikely that *Ascosphaera* would be able to infect hoverflies given their larval biology differs so much from that of bees. It is also unlikely that *Ascosphaera* infects bumblebees given that it has never been recorded parasitizing them in spite of the intensive research into their host-parasite interactions [33], [65]. It therefore seems most probable that the high incidence of *Ascosphaera* in our samples of adult pollinators reflects vectoring of the fungal spores, either on the body surface of the insects or, more probably given our sampling protocol, in their guts. The results suggest that *Ascosphaera* is very common in the environment of pollinators and that vectoring by non-host pollinators may have an important role to play in its movement around the environment.

Microsporidian parasites were relatively rare in our samples. They were most frequently found in bumblebees, with only a small number of honey bees, wasps, *Lasioglossum* bees and hoverflies also being positive for Microsporidia, and the remaining pollinators all being negative. In the case of bumblebees, the Microsporidia were *Nosema bombi*, but the identity of the microsporidians in the other pollinators is unknown. Microsporidia are common parasites in both honey bees and bumblebees, and the incidence in our samples was lower than has often been found previously [27], [33]. However, there can be substantial seasonal or population variation in the incidence of microsporidian parasites, which may explain the low levels we found [33], [66], [67].

Possibly the most important result was the discovery that DWV was present in a third of *Bombus terrestris* bumblebees and *Vespula vulgaris* wasps, as well as a few *B. pascuorum*. Symptomatic DWV infections in honey bees result in bees developing with deformed wings and thus being unable to fly, although most infections do not result in such obvious symptoms [27]. DWV has previously been found infecting, and causing symptoms in, a colony of *B. pascuorum* in Germany, as well as possibly in 10% of commercially-reared *B. terrestris* [39]. A similar molecular screening to ours detected it in every bee and wasp species examined [41], although the limited sampling in that study gave little information on prevalence. Our results indicate that DWV is in fact quite widespread in bumblebees, at least in *B. terrestris*, and is also common in *V. vulgaris* wasps. As our samples were of foraging bees and wasps collected at flowers or in pan traps, the DWV clearly had not caused the deformed wing symptoms. It therefore represented either asymptomatic infections or was being vectored. Further work will be needed to establish whether DWV is a natural parasite of these species, or has spilled over from honey bees, or was simply being vectored by them. We sampled only very few individuals for the other bumblebee and wasp species, and so the lack of DWV in these species may be due to the low sample size, and the same may be true of the other pollinators. The bumblebees and wasps were collected at different sites from most of the other bees and hoverflies, so spatial variation could also explain the differences, although it is notable that the single bumblebee sampled in the south of England was positive for DWV. Nevertheless, the complete lack of DWV in any of the other bees or hoverflies that we sampled suggests that it is not a general parasite of pollinating insects and that bumblebees and *Vespula* wasps are more likely to be infected or contaminated with DWV than other pollinators. Unlike other pollinators, both bumblebees and *Vespula* wasps rob honey from honey bee colonies. Honey has been shown to contain infective particles of DWV [41], so this may therefore be the main route by which bumblebees and *Vespula* wasps become infected or contaminated with DWV.

These preliminary data demonstrate that a wide variety of pollinators carry *Wolbachia*, *Ascosphaera*, microsporidian and DWV parasites. Regardless of whether the results represent vectoring or infection, it appears there may be significant interaction between host species in the movement of parasites. All of these parasites have the potential to substantially reduce host fitness and the results thus emphasise the importance of determining the diversity and impact of parasites in order to inform the conservation of pollinator populations. The shared use of flowers by multiple pollinator species, as well as robbing of food stores in some, has the potential to make the transmission or vectoring of parasites between taxa relatively frequent. Incorporating multi-species pollinator interactions will therefore be essential to accurately model and predict the population-level dynamics of pollinator parasites.

## Materials and Methods

### Sample collection

**Figure 2:**
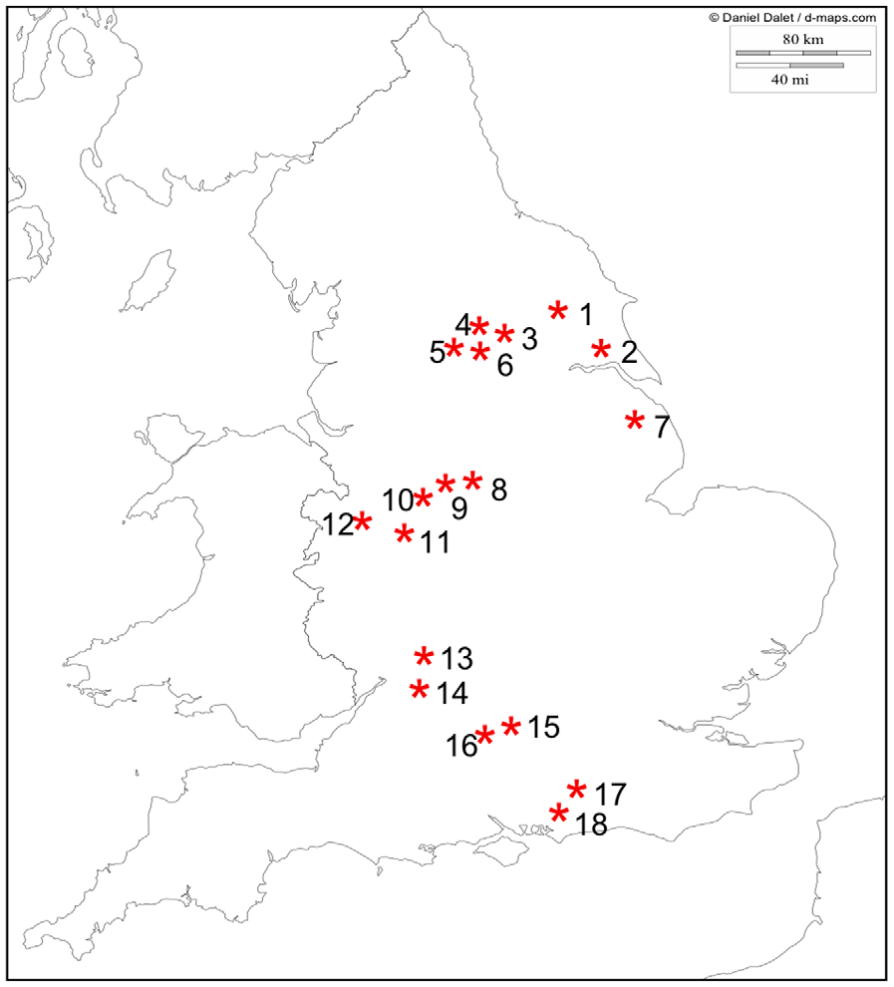
Locations of the sites at which pollinator samples were collected. Samples were collected within a 5 km area at each site. See Table 1 for the precise location of each of the numbered sites and the numbers of pollinators collected at each.

A total of 325 pollinators were collected by hand from flowers and by pan-trapping from 83 locations within 18 urban and arable sites (up to 5 km^2^) across the UK, during June/July 2007 and 2008 (Figure 2, Table S1). Hand-collected samples were stored immediately in 96% ethanol. Pan traps were checked every 48 h and samples then transferred to 96% ethanol. All samples were stored at −20°C. The samples collected included representatives of five species of bumblebees (*Bombus terrestris*, *B. pascuorum*, *B. lapidarius*, *B. pratorum*, *B. hortorum*), honey bees (*Apis mellifera*), three genera of solitary bees (*Andrena*, *Halictus*, *Lasioglossum*), three species of social wasps (*Vespula vulgaris*, *V. germanica*, *Dolichovespula sylvestris*) and four species of hoverflies (*Eristalis arbustorum*, *E. tenax*, *Episyrphus balteatus*, *Rhingia campestris*). The species of white-tailed bumblebee cannot be distinguished morphologically, so we sequenced the host CO1 gene for a subset of these individuals from each site. We compared their sequences to sequences in Genbank using BLASTN and found that all were *B. terrestris*.

### Molecular analysis

The midgut, ovaries and fat bodies were dissected out from the pollinators, as these seemed the tissues most likely to contain the parasites of interest. *Wolbachia* is generally considered a vertically transmitting symbiont so is most likely to be found in ovaries, although it can also occur at high intensities in the fat body and other tissues [22]. Microsporidia infect via the faecal-oral route, while the viruses and the *Ascosphaera* fungi also infect via ingestion, making them appear most likely to be found in the gut [27]. A small sample of the three tissues for each individual was combined, rehydrated in ddH_2_0 and homogenised using a sterile pestle. DNA and RNA was extracted by boiling the sample in 5% Chelex solution for 15 minutes. Samples were then centrifuged at 3500 rpm (1060 g) for 10 minutes and the supernatant stored at −20°C. PCR amplification of the DNA was carried out using ABI 3700 thermal cyclers in 10 µl volumes containing 1 µl DNA, 0.2 µl of each forward and reverse primer, 2 µl PCR buffer and 0.05 µl of 5 U/µl Taq (Promega). Reactions contained primer specific quantities of 25 mM MgCl_2_ and 10 mM dNTPs and made up to 10 µl with ddH_2_0. Samples were amplified for: 1) The CO1 host control gene with *LCO-Hym*/*HCOout* primers [68], [69] using 1.5 µl MgCl_2_ and 1 µl dNTPs, with an initial denaturation of 2 min at 94°C followed by 35 cycles of 30 s at 94°C, 45 s at 50°C and 2 min at 72°C, and a final extension step of 72°C for 7 min. 2) Microsporidia with the *V1f/530r* primers [70] using 1.5 µl MgCl_2_ and 0.5 µl dNTPs, with an initial denaturation of 1 min at 95°C followed by 35 cycles of 1 min at 95°C, 1 min at 60°C and 1 min at 72°C, and a final extension step of 72°C for 7 min. 3) *Ascosphaera* fungi with the *AscoAll1/AscoAll2* primers [71] using 1 µl MgCl_2_ and 1.5 µl dNTPs, with an initial denaturation of 10 min at 94°C followed by 30 cycles of 45 s at 94°C, 45 s at 62°C and 1 minute at 72°C, and a final extension step of 72°C for 5 min. 4) *Wolbachia* intracellular bacteria with *CoxA f/r* primers [72] using 1 µl MgCl_2_ and 1 µl dNTPs, with an initial denaturation of 2 min at 94°C followed by 30 cycles of 30 s at 94°C, 45 s at 55°C and 2 min at 72°C, and ending with a final extension step of 72°C for 7 min. PCR products were visualised under UV using 1% agarose gels stained with ethidium bromide and compared to a 100 bp size ladder. Positive and negative controls were included in every PCR. The DNA from a subset of positive samples were subsequently amplified in 50 µl PCR reactions and purified using the Qiaquick PCR purification kit (Qiagen). Products were sequenced using the ABI Dye Terminator Labelled Sequencing sytem with an ABI3030*xl* capillary sequencer, and the resulting sequences compared with existing sequences in Genbank using a BLASTN search.

The Chelex extractions of all samples were in addition screened for the presence of six honey bee viruses using Taqman real-time PCR assays (Table 1). Probes for the detection of black queen cell virus (BQCV), chronic bee paralysis virus (CBPV), deformed wing virus (DWV) and Israeli acute paralysis virus (IAPV) were duel labelled with the fluorescent reporter dye FAM (6-carboxyfluorescein) at the 5′ end and with the fluorescent quencher dye TAMRA at the 3′ end. Probes for the detection of acute bee paralysis virus (ABPV) and sacbrood virus (SBV) substituted TAMRA for a minor groove binder (MGB) at the 3′ end. Each sample was screened in duplicate 25 µl reactions comprising 10× Buffer A (50 mMKCl, 10 mM Tris–HCl, pH 8.3, carboxy-X-rhodamine [ROX] passive reference dye), 0.025 U AmpliTaq Gold, 0.2 mM (each) deoxynucleoside triphosphate, 5.5 mM MgCl_2_, 0.016 U MMLV, 300 nM of each primer and 100 nM of duel-labelled probe. Reactions were run on 384-well plates and cycled using generic system conditions (48°C for 30 min, 95°C for 10 min and 40 cycles of 60°C for 1 min plus 95°C for 15 s) within the 7900 Sequence Detection System (Applied Biosystems, Branchburg, New Jersey, USA) using real-time data collection. Positive and negative controls were included in all real-time PCR assays. It should be noted that while the Chelex method has been used successfully for extraction of RNA viruses [73], it is of limited effectiveness [45], [46], and so our protocol may underestimate the true levels of viruses present.

**Table 1:** Real-time Taqman PCR primers and probes used to detect acute bee paralysis virus (ABPV), black queen cell virus (BQCV), chronic bee paralysis virus (CBPV), deformed wing virus (DWV), Israeli acute paralysis virus (IAPV) and sacbrood virus (SBV).

Virus	Forward primer	Reverse primer	Probe
**ABPV**	*ABPV 5436F*:TAA CCA ATG AAG TRT CCA TAG GAA CTA	*ABPV 5481R*:TCT CCT GCR ATA ACC TTG GGT	*ABPV 5515TMGB*:TGT TTA TTC CCA AGA TTG
**BQCV** 1 **^,^****** 3	*BQCV 9195F*:GGT GCG GGA GAT GAT ATG GA	*BQCV 8265R*:GCC GTC TGA GAT GCA TGA ATA C	*BQCV 8217T*:TTT CCA TCT TTA TCG GTA CGC CGC C
**CBPV** 2 **^,^** 3	*CBPVF*:CGC AAG TAC GCC TTG ATA AAG AAC	*CBPVR*:ACT ACT AGA AAC TCG TCG CTT CG	*CBPVT*:TCA AGA ACG AGA CCA CCG CCA AGT TC
**DWV** 1 **^,^** 3	*DWV 9587F*:CCT GGA CAA GGT CTC GGT AGA A	*DWV 9711R*:ATT CAG GAC CCC ACC CAA AT	*DWV 9627T*:CAT GCT CGA GGA TTG GGT CGT CGT
**IAPV** 2 **^,^** 3	*IAPV B4S0427_R130M*:RCR TCA GTC GTC TTC CAG GT	*IAPV B4S0427_L17M*:CGA ACT TGG TGA CTT GAR GG	*IAPVT*:TTG CGG CAA TCC AGC CGT GAA AC
**SBV** 2 **^,^** 3	*SBV 311F*:AAG TTG GAG GCG CGY AAT TG	*SBV 380R*:CAA ATG TCT TCT TAC DAG AGG YAA GGA TTG	*SBV 331TMGB*:CGG AGT GGA AAG AT

1 [74].

2 [75].

3 [76].

### Statistical analysis

We analysed our data using Generalized Linear Models for binomially distributed data with a logit link function. For each parasite, we compared the numbers of samples that were positive and negative between the five pollinator types (honey bees, bumblebees, other bees, wasps and hoverflies). In addition, we compared the occurrence of parasites between host species within each host type, to determine whether certain species were more likely to have the parasite than others.

### Ethics

No specific permits were required for the described field studies. Permission to collect samples on their private land was provided by Tom Cameron, Rebecca Neal, Alex Bateman, Kenneth McDowall, Jane Device, Iain Manfield, Will Patterson, Thomas Edwards, Paul Drake, Dick Hobson, Stan Burgess, Andy Ford, Lars Jeuken, Mark Harris, Hayley Lynch, Jenny Dunn, John Illingworth, Sam Mason, Paul Millner, Neal Haddaway, Alison Dunn, Terry McAndrew, Peter Henderson, Dave Adams, Carol Davison, Ben Chapman, Brenda Frater, Steffi Jourdan, Tim Johnson, Nicky Spencer Jones, Lesley Hooper, Anne Proud, Emma Black, Fiona Moulton, Teegan Docherty, Roberta Pagliarini, Pat Shore, Liz Paget, Richard Rodway and James Rosindell. All other samples were collected on public land that was not protected in any way and for which no specific permissions were required. The field studies did not involve endangered or protected species.

## Supporting Information

Table S1 Details of the samples collected and parasites found, including map point (referring to Figure 1), location and date of sampling, numbers of individuals of each host species collected at each site, and numbers of each of these species that were found by molecular screening to be positive for each of the parasites.
(DOCX)
