# Supplementary material for: Pervasiveness of Parasites in Pollinators
Source: PLoS One. 2012 Jan 26;7(1):e30641. doi: 10.1371/journal.pone.0030641 (PMC3273957; doi:10.1371/journal.pone.0030641)
Supplement: Table S1 — Details of the samples collected and parasites found, including map point (referring to Figure 1), location and date of sampling, numbers of individuals of each host species collected at each site, and numbers of each of these species that were found by molecular screening to be positive for each of the parasites. (DOCX) [file pone.0030641.s001.docx]

**Supporting Information**

Table S1. Details of the samples collected and parasites found, including map point (referring to Figure 1), location and date of sampling, numbers of individuals of each host species collected at each site, and numbers of each of these species that were found by molecular screening to be positive for each of the parasites.

| **Map point** | **Location** | **Sampling date** | **Species collected (# individuals)** | **Parasites found (# individuals)** |
| --- | --- | --- | --- | --- |
| 1 | Sand Hutton, | July 2008 | *Bombus lapidarius* (1) | *Wolbachia* (1) |
|  | North Yorkshire |  | *Bombus pascuorum* (8) | *Wolbachia* (8); DWV (2) |
|  |  |  | *Bombus terrestris* (3) | *Wolbachia* (2); DWV (2) |
|  |  |  | *Vespula vulgaris* (12) | Microsporidia (5); *Wolbachia* (5); *Ascosphaera* (9); DWV (6); BQCV (1); SBV (1) |
| 2 | Riplingham, | June/July 2008 | Andrena (1) | *Ascosphaera* (1) |
|  | East Yorkshire |  | *Episyrphus balteatus* (2) | *Wolbachia* (2); *Ascosphaera* (2) |
|  |  |  | Unidentified solitary bee (1) | *Ascosphaera* (1) |
| 3 | Roundhay, Leeds, | July 2008 | *Bombus hortorum* (4) | Microsporidia (1); *Wolbachia* (4); *Ascosphaera* (4) |
|  | West Yorkshire |  | *Bombus lapidarius* (2) | Microsporidia (1); *Wolbachia* (1); *Ascosphaera* (2) |
|  |  |  | *Bombus pratorum* (2) | *Wolbachia* (2); *Ascosphaera* (2) |
|  |  |  | *Bombus terrestris* (3) | Microsporidia (1); *Wolbachia* (2); *Ascosphaera* (2) |
|  |  |  | *Vespula vulgaris* (3) | *Wolbachia* (3); *Ascosphaera* (3) |
| 4 | Harewood Estate, | September 2008 | *Apis mellifera* (7) | DWV (7) |
|  | West Yorkshire |  | *Bombus pascuorum* (20) | Microsporidia (10); *Wolbachia* (20) |
|  |  |  | *Bombus terrestris* (46) | Microsporidia (3); *Wolbachia* (46); *Ascosphaera* (2); DWV (14) |
|  |  |  | *Vespula germanica* (1) | DWV (1) |
|  |  |  | *Vespula vulgaris* (8) | Microsporidia (1); DWV (8) |
| 5 | Horsforth, Leeds, | July 2008 | *Bombus hortorum* (1) | *Wolbachia* (1); *Ascosphaera* (1) |
|  | West Yorkshire |  | *Bombus pratorum* (3) | *Wolbachia* (2); *Ascosphaera* (2) |
|  |  |  | *Bombus terrestris* (4) | Microsporidia (1); *Wolbachia* (3); *Ascosphaera* (4) |
|  |  |  | *Vespula vulgaris* (2) | *Wolbachia* (1); *Ascosphaera* (1) |
| 6 | Headingly, Leeds, | July 2008 | *Bombus hortorum* (3) | Microsporidia (2); *Wolbachia* (1); *Ascosphaera* (1) |
|  | West Yorkshire |  | *Bombus lapidarius* (2) | Microsporidia (1); *Wolbachia* (2); *Ascosphaera* (2) |
|  |  |  | *Bombus pascuorum* (8) | Microsporidia (4); *Wolbachia* (5); *Ascosphaera* (8) |
|  |  |  | *Bombus pratorum* (3) | Microsporidia (1); *Wolbachia* (1); *Ascosphaera* (3) |
|  |  |  | *Bombus terrestris* (1) | *Ascosphaera* (1) |
|  |  |  | *Vespula vulgaris* (1) | No parasites found |
| 7 | Biscathorpe, | July 2008 | *Andrena* (4) | *Wolbachia* (3); *Ascosphaera* (4) |
|  | Lincolnshire |  | *Dolichovespula sylvestris* (1) | *Wolbachia* (1) |
|  |  |  | *Episyrphus balteatus* (1) | *Wolbachia* (1); *Ascosphaera* (1) |
|  |  |  | Unidentified solitary bee (2) | *Wolbachia* (2); *Ascosphaera* (2) |
|  |  |  | *Vespula vulgaris* (6) | *Wolbachia* (2); *Ascosphaera* (3) |
| 8 | Rodsley, | June/July 2007, | *Vespula vulgaris* (2) | *Wolbachia* (2); *Ascosphaera* (2) |
|  | Derbyshire | July 2008 | *Eristalis tenax* (1) | No parasites found |
| 9 | Rocester, | July 2007 | *Eristalis arbustorum* (1) | *Wolbachia* (1); *Ascosphaera* (1) |
|  | Staffordshire |  | *Eristalis tenax* (3) | No parasites found |
| 10 | Oulton, | June 2007, | *Eristalis arbustorum* (10) | *Wolbachia* (7); *Ascosphaera* (9) |
|  | Staffordshire | July 2008 | *Eristalis tenax* (3) | *Wolbachia* (1); *Ascosphaera* (1) |
|  |  |  | *Rhingia campestris* (10) | *Wolbachia* (4); *Ascosphaera* (2) |
|  |  |  | *Vespula germanica* (1) | No parasites found |
| 11 | Orslow, | June/July 2007, | *Episyrphus balteatus* (2) | *Wolbachia* (1); *Ascosphaera* (1) |
|  | Staffordshire | July 2008 | *Eristalis tenax* (2) | Microsporidia (2); *Wolbachia* (2); *Ascosphaera* (1) |
|  |  |  | *Lassioglossum* (1) | No parasites found |
|  |  |  | *Vespula vulgaris* (1) | No parasites found |
| 12 | Merrington, | June/July 2007, | *Episyrphus balteatus* (4) | *Wolbachia* (3); *Ascosphaera* (3) |
|  | Shropshire | July 2008 | *Eristalis tenax* (2) | *Wolbachia* (2); *Ascosphaera* (2) |
|  |  |  | *Rhingia campestris* (2) | *Wolbachia* (1) |
|  |  |  | *Vespula germanica* (1) | *Wolbachia* (1); *Ascosphaera* (1) |
|  |  |  | *Vespula vulgaris* (2) | *Wolbachia* (2); *Ascosphaera* (2) |
| 13 | Gloucester, | June/July 2008 | *Episyrphus balteatus* (1) | *Wolbachia* (1); *Ascosphaera* (1) |
|  | Gloucestershire |  | *Lassioglossum* (1) | No parasites found |
| 14 | Tetbury, | July 2008 | *Andrena* (1) | *Wolbachia* (1); *Ascosphaera* (1) |
|  | Gloucestershire |  | *Episyrphus balteatus* (1) | *Wolbachia* (1); *Ascosphaera* (1) |
|  |  |  | *Lasioglossum* (4) | *Wolbachia* (1); *Ascosphaera* (1) |
|  |  |  | *Vespula vulgaris* (1) | *Ascosphaera* (1) |
| 15 | Fosbury, | July 2008 | *Episyrphus balteatus* (2) | *Wolbachia* (1) |
|  | Wiltshire |  | *Lasioglossum* (3) | Microsporidia (1); *Ascosphaera* (2) |
|  |  |  | Unidentified solitary bee (2) | No parasites found |
|  |  |  | *Vespula vulgaris* (1) | *Wolbachia* (1); *Ascosphaera* (1) |
| 16 | East Chisenbury, Wiltshire | June/July 2008 | *Lassioglossum* (4) | No parasites found |
| 17 | Eastbourne, | July 2008 | *Andrena* (2) | No parasites found |
|  | West Sussex |  | *Episyrphus balteatus* (1) | *Wolbachia* (1); *Ascosphaera* (1) |
|  |  |  | *Halictus* (1) | *Ascosphaera* (1) |
|  |  |  | *Lasioglossum* (6) | *Wolbachia* (1); *Ascosphaera* (3) |
|  |  |  | *Vespula vulgaris* (5) | *Ascosphaera* (2) |
|  |  |  | Unidentified solitary bee (10) | *Wolbachia* (1); *Ascosphaera* (2) |
| 18 | Chichester, | June 2007, | *Andrena* (5) | *Wolbachia* (5); *Ascosphaera* (5) |
|  | West Sussex | July 2008 | *Apis mellifera* (5) | Microsporidia (1); *Wolbachia* (2); *Ascosphaera* (2); DWV (5) |
|  |  |  | *Bombus terrestris* (1) | DWV (1) |
|  |  |  | *Halictus* (19) | *Wolbachia* (14); *Ascosphaera* (13) |
|  |  |  | *Lasioglossum* (26) | *Wolbachia* (15); *Ascosphaera* (14) |
|  |  |  | Unidentified solitary bee (1) | No parasites found |
|  |  |  | *Vespula germanica* (1) | No parasites found |
|  |  |  | *Vespula vulgaris* (4) | *Wolbachia* (1); *Ascosphaera* (1) |
